# β-Adrenoceptor Activation in Breast MCF-10A Cells Induces a Pattern of Catecholamine Production Similar to that of Tumorigenic MCF-7 Cells

**DOI:** 10.3390/ijms21217968

**Published:** 2020-10-27

**Authors:** Filipa Amaro, Dany Silva, Henrique Reguengo, José C. Oliveira, Clara Quintas, Nuno Vale, Jorge Gonçalves, Paula Fresco

**Affiliations:** 1 Laboratory of Pharmacology, Department of Drug Sciences, Faculty of Pharmacy, University of Porto, 4050-313 Porto, Portugal; famaro@ff.up.pt (F.A.); up201708266@fc.up.pt (D.S.); claraquintas@ff.up.pt (C.Q.); nuno.vale@ff.up.pt (N.V.); pfresco@ff.up.pt (P.F.); 2 Clinical Chemistry, Department of Laboratory Pathology, Centro Hospitalar Universitário do Porto (CHUP), 4099-001 Porto, Portugal; henrique.reguengo.sqc@chporto.min-saude.pt (H.R.); director.sqc@chporto.min-saude.pt (J.C.O.); 3 Unit for Multidisciplinary Research in Biomedicine (UMIB), Institute of Biomedical Sciences Abel Salazar (ICBAS), University of Porto, 4050-313 Porto, Portugal; 4 Epithelial Interactions in Cancer, I3S, University of Porto, 4200-135 Porto, Portugal

**Keywords:** breast cancer, β-adrenoceptors, catecholamine synthesis, tumorigenic process, propranolol

## Abstract

Adrenaline, which participates in the neuroendocrine response that occurs during stress and perimenopause, may be tumorigenic. This exploratory study aimed at investigating whether non-tumorigenic and tumorigenic human breast epithelial cell lines are able to synthesize adrenaline. The study was carried out in non-tumorigenic (MCF-10A) and tumorigenic (MCF-7) human breast cell lines. Expression of enzymes involved in adrenaline synthesis was characterized by RT-qPCR, immunocytochemistry and western blot. Catecholamines and analogue compounds were quantified by HPLC-ECD. Functional assessment of the impact of drugs on cells’ tumorigenic potential was assessed by determination of cell viability and clonogenic ability. Both MCF-10A and MCF-7 cells produce catecholamines, but the capacity to produce adrenaline is lower in MCF-10A cells. β-adrenoceptor activation increases the capacity of MCF-10A cells to produce adrenaline and favor both cell viability and colony formation. It is concluded that exposure of human breast epithelial cells to β-adrenoceptor agonists increases cell proliferation and the capacity to produce adrenaline, creating an autocrine potential to spread these adrenergic effects in a feed-forward loop. It is conceivable that these effects are related to tumorigenesis, bringing a new perspective to understand the claimed anticancer effects of propranolol and the increase in breast cancer incidence caused by stress or during perimenopause.

## 1. Introduction

Exposure to major stressful events is associated with higher breast cancer risk [1,2] and with lower survival rates in patients with breast cancer [3]. The main stress response mechanisms are the activation of the hypothalamic–pituitary–adrenal axis, with a consequent increase in plasmatic cortisol levels and the activation of the sympathetic nervous system (SNS). SNS activation is accompanied by a long-lasting increase of the catecholamines, noradrenaline, and adrenaline, in plasma [4]. An increase in plasma catecholamines also occur during perimenopause, which seems to contribute to the cardiovascular changes accompanying hot flashes [5,6]. Noradrenaline and adrenaline orchestrate a *fight or flight* response (increased cardiac output, energy mobilization to the periphery, increased awareness state) typical of the reaction to acute stress. They may also promote other types of biological processes, some favorable to tumorigenesis/carcinogenesis, namely stimulation of cancer cell proliferation [7] and inhibition of immune surveillance [8,9]. 

The first evidence of a link between adrenergic stimulation and carcinogenesis dates back to the middle of last century, after the observation that local injection of adrenaline increased the rate of tumor formation [10]. Further studies corroborated this link by showing that adrenergic stimulation induces proliferation of colon cancer cells [11], growth of colorectal carcinoma in vivo [12], and migration of carcinoma cells from colon [13], nasopharynx [14], prostate [15] and pancreas [16].

Adrenaline and noradrenaline effects are mediated by a family of G-protein coupled receptors, named adrenoceptors [17]. Adrenoceptors are subdivided into three major types (α_1_, α_2_, and β), each further divided into three subtypes (α_1A_, α_1B_, α_1D_; α_2A_, α_2B_, α_2C_; and β_1_, β_2_, and β_3_). Adrenoceptors from the three types (α_1,_ α_2,_ and β) are involved in the adrenergic modulation of carcinogenesis [9,18]. The type involved in this modulation may differ according to the tissue: α_1_-adrenoceptors increase proliferation of gastric [19] and prostate [20] cancer cells; α_2_-adrenoceptors increase proliferation of breast cancer cells [21,22], whereas β-adrenoceptors, mainly β_2_, seem to increase cell proliferation of lung [23,24], breast [25,26], ovarian [27], pancreas [28], colon [11] cancer and of melanoma cells [9,29]. Observational studies have shown that blockade of β-adrenoceptors increase overall survival in cancer patients [30,31], indicating the existence of β-adrenoceptor-mediated effects with clinical relevance in the modulation of carcinogenesis. 

It is generally assumed that the adrenoceptor endogenous agonists, noradrenaline, and adrenaline, involved in tumor regulation have a neuroendocrine origin, and that β-blockers would reduce cancer mortality by blocking a putative carcinogenic effect of the catecholamines released from the sympathetic nerves or from the adrenal medulla [32,33]. Although tumors may recruit sympathetic adrenergic nerves [34], the possibility that tumor cells may also contribute to the catecholamine pool must also be considered. It was recently reported that human breast epithelial cells express tyrosine hydroxylase (TH; the rate-limiting enzyme of catecholamine synthesis) and produce noradrenaline, described as a putative mechanism for the stress-induced changes in milk composition [35]. Moreover, transfected breast tumorigenic cells (MCF-7 overexpressing Her-2) were also reported to produce catecholamines [36]. The possibility that catecholamines involved in carcinogenesis could be formed locally deserves to be further explored because it opens the hypothesis that, at least in breast tissue, the adrenergic-induced carcinogenesis may have an autocrine contribution. In the present study, this hypothesis was explored by investigating whether breast cells that differ in their tumorigenic potential (non-tumorigenic MCF-10A, and tumorigenic MCF-7 cells), (i) possess the ability to synthesize catecholamines, (ii) if there are differences in the synthetic ability according to the cell tumorigenic potential and (iii) if this ability is influenced by exogenous β-adrenoceptor ligands (to clarify a putative contribution of this mechanism for the reduction of breast cancer incidence and mortality caused by β-blockers).

## 2. Results

### 2.1. Expression of Enzymes Involved in the Biosynthesis of Catecholamines

To address whether human breast non-tumorigenic MCF-10A and tumorigenic MCF-7 cells express the enzymatic machinery responsible for the biosynthesis of catecholamines, expression of TH (the rate-limiting enzyme of catecholamine synthesis) and phenylethanolamine N-methyltransferase (PNMT, the enzyme that converts noradrenaline to adrenaline), was investigated in these two cell lines by RT-qPCR and by immunocytochemistry.

**Figure 1:**
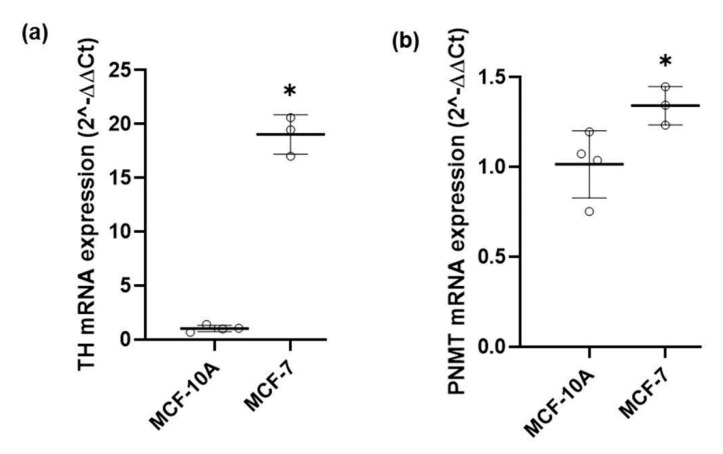
mRNA expression of (**a**) tyrosine hydroxylase (TH) and (**b**) phenylethanolamine N-methyltransferase (PNMT), in MCF-10A and MCF-7 cells, determined using RT-qPCR and normalized to β-actin (Ct values for β-actin are available in Figure S1). Similar results were obtained when normalized to GAPDH. Values are means ± SD from 3 (MCF-10A cells) or 4 (MCF-7 cells) independent experiments. Significant differences from TH or PNMT expression in MCF-10A cells: * *p* ≤ 0.05 (Student’s *t*-test).

As shown (Figure 1), mRNA for TH and PNMT was found both in MCF-10A and in MCF-7 cells indicating the existence of a transcription process operating for the TH and PNMT genes in both cell lines. However, there was a marked difference in the two cell lines: the mRNA levels of TH were much higher in the tumorigenic MCF-7 cells, compared to that observed in the non-tumorigenic MCF-10A cells (Figure 1a); mRNA levels of PNMT were also higher in MCF-7 cells (Figure 1b) but much lower than those of TH in both cell lines.

**Figure 2:**
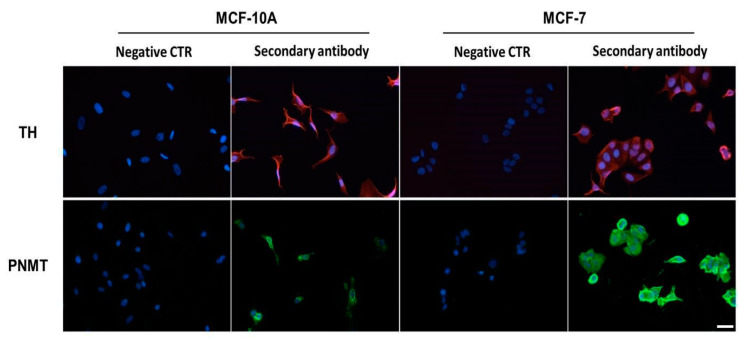
Protein expression of tyrosine hydroxylase (TH) and phenylethanolamine N-methyltransferase (PNMT) in MCF-10A or in MCF-7 cells, revealed by immunocytochemistry. Shown are representative microphotographs of immunoreactivities revealed by secondary antibodies conjugated with Alexa fluor 594 (red fluorescence, TH) or with Alexa fluor 488 (green fluorescence, PNMT). Nuclei were labelled with Hoechst 33342 (blue fluorescence). Scale bar: 20 µm. Negative control (CTR): without primary antibody.

Expression of TH and PNMT was further proceeded to investigate protein expression by immunocytochemistry. Both MCF-10A and MCF-7 cells presented immunoreactivities for anti-TH and anti-PNMT antibodies, absent in the negative controls (Figure 2), indicating that transcription proceeds to the translation of both enzymes (TH and PNMT) in the two cell lines.

### 2.2. Catecholamines Biosynthesis

The ability of cells to synthesize catecholamines was assessed by looking for the presence of endogenous catecholamines, both in the cellular fraction and in the conditioned medium of both cell lines. 

This ability was further assessed by incubating the cells with an exogenous substrate (α-methylDOPA). Although not dependent on TH, α-methylDOPA can be metabolized by the enzymes of the catecholamine biosynthesis pathway (DOPA decarboxylase, dopamine β-hydroxylase and PNMT). Therefore, the presence of α-methyl derivatives of endogenous catecholamines in the cell’s conditioned media may be taken as a strong indicator of a fully operating system in these cell lines. The endogenous catecholamines and α-methylDOPA metabolites were detected by LC-MS (Figure S2) and/or quantified by HPLC-ECD. Culture media, without cells, were kept in the same incubation conditions and used as controls.

In the cellular fraction of MCF-10A or MCF-7 cells, as well as in medium without cells (controls), noradrenaline and adrenaline were not detected. However, both catecholamines were present in the conditioned medium of both cell lines (Table 1). The MCF-7 cells adrenaline/noradrenaline ratio was higher than that of MCF-10A cells (about 5.8 and 0.9, respectively), indicating that MCF-7 cells produce mainly adrenaline.

To confirm the presence of an operational catecholamine synthetic pathway in MCF-10A and MCF-7 cells, both cell lines were exposed to 10 or 100 µM α-methylDOPA. After a 24-h exposure of cells to α-methylDOPA, this pseudo-substrate was no longer detected in the conditioned medium of either MCF-10A or MCF-7 cells. However, its metabolite (α-methylnoradrenaline) was detected in the conditioned media of both cell lines and the concentration of α-methylnoradrenaline was even higher when cells were exposed to the higher concentration (100 µM) of α-methylDOPA (Table 2). LC-MS assays confirmed the presence of chemical entities with molecular weights corresponding to successive methylations of α-methylnoradrenaline in the conditioned medium of MCF-7 cells (Figure S2). 

The lower concentration of α-methylnoradrenaline in the conditioned medium of MCF-7 cells, comparatively to that of MCF-10A cells initially exposed to the same concentration of α-methylDOPA (10 or 100 µM), suggests that the metabolization of α-methylnoradrenaline into α-methyladrenaline occurs at a higher rate in MCF-7 cells (Table 2). This finding is in agreement with the higher PNMT mRNA expression found in MCF-7 cells (Figure 1). Taken together, these results provide additional evidence to support the existence of an operational catecholamine biosynthetic pathway in both cell lines, albeit more efficient in MCF-7 cells. 

### 2.3. Influence of β-Adrenergic Receptor Activation

Increased cell viability and colony formation are parameters accepted to be hallmarks of tumorigenesis [37]. Therefore, the MTT and the clonogenic assays were used to explore the effects of adrenoceptor activation in both MCF-10A and MCF-7 cells. Because of the recognized protective effects of β-blockers in breast cancer [31] and of the role of β_2_-adrenoceptors in the modulation of breast cancer cell proliferation [25,26], the experimental approach was designed to target β_2_-adrenoceptor, which expression was confirmed in both MCF-10A and MCF-7 cell lines (Figure S3). Pharmacological interference with the β-adrenoceptors was based on the use of the β-adrenoceptor agonist, isoprenaline, and on the β-adrenoceptor antagonists, propranolol (non-selective) and ICI 118,551 (selective for the β_2_-adrenoceptor subtype). 

#### 2.3.1. Cell Viability

**Figure 3:**
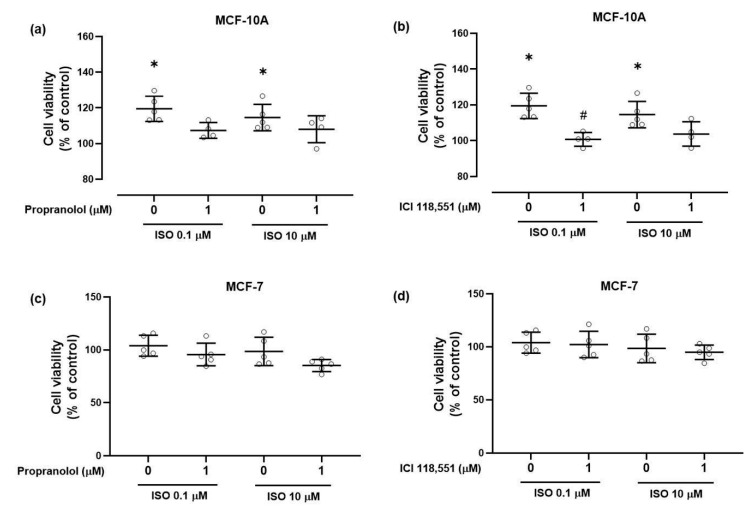
Influence of β-adrenoceptor agonist, isoprenaline (ISO, 0.1 and 10 µM), alone or in combination with β-adrenoceptor antagonists, (**a**,**c**) propranolol (1 µM) or (**b**,**d**) ICI 118,551 (1 µM) on cell viability of (**a**,**b**) MCF-10A and (**c**,**d**) MCF-7 cells, using the MTT assay. Propranolol and ICI 118,551, *per se*, did not change cell viability in both cell lines tested (see text). Cells were treated with the indicated drugs for 24 h. Results are expressed as percentage of control (solvent) and are presented as mean ± SD. Values are means ± SD from 4–5 (MCF-10A cells) or 5 (MCF-7 cells) independent experiments, as shown. Significant differences from control group: * *p* ≤ 0.05; one-way analysis of variance (ANOVA), followed by post-hoc multi-comparisons Dunnett’s test. Significant differences from isoprenaline treatment: * *p* ≤ 0.05; (Student’s *t*-test).

The effects of the adrenergic ligands in the viability of non-tumorigenic MCF-10A cells are shown in Figure 3. After a 24-h exposure period, isoprenaline (0.1, 10 μM) increased the viability of MCF-10A cells (Figure 3). Propranolol or ICI 118,551 (1 μM), per se, had no effect on cell viability (98.9 ± 10.4% and 106.8 ± 4.8%; *n* = 4, respectively) even when high concentrations (100 μM) were used (101.6 ± 13.7% and 100.8 ± 6.2%; *n* = 5, respectively). Cell viability was also not altered by prazosin (1 µM) or by yohimbine (1 µM), antagonists of α_1_- and α_2_-adrenoceptors, respectively.

When combined with isoprenaline (0.1, 10 μM), both propranolol (Figure 3a) and ICI 118,551 (Figure 3b) prevented the increase in cell viability caused by isoprenaline. Since ICI 118,551 is a selective antagonist for the β_2_-adrenoceptor subtype, these results indicate a putative involvement of the β_2_-adrenoceptor subtype in the increase of the viability of MCF-10A cells. 

In MCF-7 cells, isoprenaline (0.1, 10 μM) exposure during 24 h did not change cell viability (Figure 3c,d). The β-antagonists propranolol or ICI 118,551 (1 μM), per se, had no effect on cell viability (91.6 ± 15.6% and 101.6 ± 20.5%; *n* = 5, respectively) and did not alter the effects of isoprenaline after a 24-h exposure (Figure 3c,d). The α_1_-adrenoceptor antagonist prazosin (1 µM) also did not alter cell viability. However, yohimbine (1 µM), the α_2_-adrenoceptor antagonist, reduced cell viability (to 81.2 ± 7.1%; *n* = 5; *p* ≤ 0.05).

An increase of the incubation time from 24 to 72 h was able to reveal an inhibitory effect of 10 µM isoprenaline (to 59.8 ± 2.9%; *n* = 4; *p* ≤ 0.05). Inhibition of cell viability was also observed when the concentration of propranolol was increased to 100 µM and the incubation time kept at 24 h (to 54.3 ± 14.0%; *n* = 5; *p* ≤ 0.05). A similar effect was observed with 100 µM ICI 118,551 (to 31.3 ± 2.5%; *n* = 5; *p* ≤ 0.05). 

Taken together, these results show that adrenoceptors influence the viability of the tumorigenic MCF-7 cells in a manner distinct from that of the non-tumorigenic MCF-10A cells, presenting and odd profile in which exogenous β-adrenoceptor agonists and antagonists seem to elicit the same type of response.

#### 2.3.2. Clonogenic Ability

**Figure 4:**
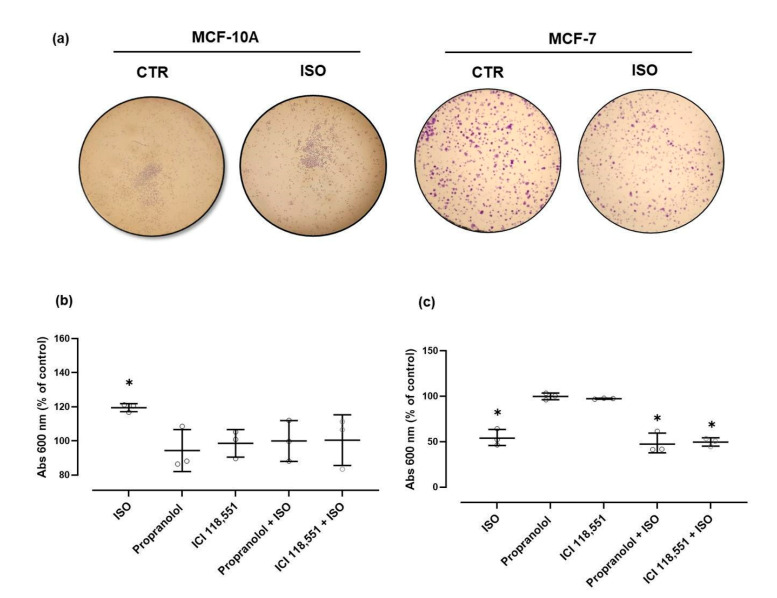
Influence of β-adrenoceptor agonist, 10 µM isoprenaline (ISO), alone or in combination with β-adrenoceptor antagonists, propranolol or ICI 118,551 (1 µM), in MCF-10A and in MCF-7 cells clonogenic ability. Cells were treated with the indicated drugs for seven days. (**a**) Representative images of the colony formation assay in MCF-10A and in MCF-7 cells in the absence or in the presence of isoprenaline (10 µM). (**b**) Results for absorbance (λ = 600 nm: Abs 600 nm), after crystal violet elution in MCF-10A cells. (**c**) Results for absorbance (λ = 600 nm: Abs 600 nm), after crystal violet elution in MCF-7 cells. Results are expressed as a percentage of control (solvent) and are presented as mean ± SD, from 3 independent experiments. Significant differences from control group: * *p* ≤ 0.05; one-way analysis of variance (ANOVA), followed by post-hoc multi-comparisons Dunnett’s test.

Further characterization of potential tumorigenic effects elicited by β-adrenergic receptor activation was performed using clonogenic assays. The effects on the number of colonies were evaluated after incubation with the indicated drugs for seven days (Figure 4). Images of a representative experiment with MCF-10A cells are shown in Figure 4a. Colony quantification showed that the β-adrenoceptor agonist isoprenaline (10 μM) markedly increased the number of colonies formed. The β-adrenoceptor antagonists, propranolol or ICI 118,551 (both at 1 μM), per se, had no effect on the number of MCF-10A colonies formed. However, when combined with isoprenaline (10 μM), both 1 µM propranolol and 1 µM ICI 118,551 prevented the increase in the number of colonies caused by isoprenaline (Figure 4b). These results are in line with those obtained in the viability assays (see Figure 3).

In MCF-7 cells, this long exposure (7 days) revealed an ability of isoprenaline to reduce colony formation: isoprenaline (10 μM) reduced the number of colonies formed (Figure 4c) whereas propranolol or ICI 118,551 (1 μM), per se, did not change the clonogenic ability of MCF-7 cells. When combined with isoprenaline (10 μM), neither propranolol nor ICI 118,551 were able to antagonise the effect of the β-adrenoceptor agonist isoprenaline. Images of a representative experiment are shown in Figure 4a.

#### 2.3.3. Catecholamine Biosynthetic Capacity

As shown in the assays described above, MCF-7 cells have an ability to synthesize adrenaline greater than that of MCF-10A cells. In order to investigate whether exposure to a β-adrenoceptor agonist could alter the ability of MCF-10A cells to synthesize adrenaline, MCF-10A cells were exposed to 0.1 µM isoprenaline and catecholamines presented in the conditioned medium were quantified. Contrasting with the profile described previously (Table 1), in these experimental conditions, noradrenaline was no longer detected in the conditioned medium of MCF-10A cells, whereas the concentration of adrenaline doubled (from 1.65 ± 0.29 nM to 3.32 ± 0.41 nM; *n* = 5; *p* ≤ 0.05), presenting an adrenaline/noradrenaline ratio closer to that of the tumorigenic MCF-7 cells. In MCF-7 cells, 0.1 µM isoprenaline doubled the concentration of noradrenaline in the conditioned medium (from 1.89 ± 0.51 nM to 3.97 ± 0.98 nM; *n* = 5; *p* ≤ 0.05) but reduced, about 75%, the concentration of adrenaline (from 11.02 ± 1.05 nM to 2.77 ± 1.33 nM; *n* = 5; *p* ≤ 0.05). 

## 3. Discussion

The catecholamines noradrenaline and adrenaline are the endogenous ligands of adrenoceptors. They participate in cellular communication, with a well-known role in the neuroendocrine stress-related fight or flight response triggered by the SNS. Therefore, responses mediated by these catecholamines (including in tumorigenesis [32,33,38] have been solely associated with the activation of the SNS. The present study shows that breast cells can also synthesize catecholamines, confirming previous observations in normal human mammary epithelial cells [35], and in non-tumorigenic and tumorigenic breast cell lines [35,36]. 

Noradrenaline and adrenaline are sequentially synthesized from tyrosine, and the conversion of tyrosine to L-DOPA, catalyzed by TH, is considered the rate-limiting step of catecholamine synthesis [39,40]. TH detection has been used to identify cells that synthesize catecholamines and the levels of TH expression as a way to estimate the potential of a given cell to produce catecholamines [35,36,41]. The present study shows that TH is expressed both in MCF-10A and MCF-7 breast cell lines. However, TH expression in tumorigenic MCF-7 cells is much higher than that observed in non-tumorigenic MCF-10A cells, suggesting that TH expression levels may be linked to the cells’ tumorigenic profile.

The TH gene has been previously reported to be a HIF regulated gene [42]. HIF-1α and HIF-2α activation induce the expression of genes that are under the control of the hypoxia-responsive element [43]. Tumorigenesis may be associated with a cellular response similar to that occurring when cells are exposed to hypoxia (pseudohypoxia). The higher expression level of TH in the tumorigenic cell line (MCF-7), observed in the present study, could be a consequence of the tumorigenic process activated by pseudohypoxia [44]. HIFs may also induce expression of DOPA decarboxylase [45], dopamine β-hydroxylase [46], and PNMT [47] enzymes which may explain the higher ability of the tumorigenic cells to synthesize catecholamines, in particular, adrenaline. 

In the present study, it is shown that relevant enzymes of the catecholamine biosynthetic pathway are expressed and are active in breast MCF-10A and MCF-7 cells, indicating that catecholamines can be produced in an autocrine way, by non-neuronal breast cells, as revealed by i) the detection of catecholamines in the conditioned media of both cell lines and by ii) their capacity to metabolize α-methylDOPA to α-methylnoradrenaline, an approach previously used in other experimental models and in vivo [48]. Therefore, the described adrenergic effects on breast cancer [49,50,51] should take into consideration sources of noradrenaline and adrenaline other than the neuroendocrine-released catecholamines.

In non-tumorigenic human breast cells, the main catecholamine produced was reported to be noradrenaline [35]. In the present study, in the tumorigenic MCF-7 cells, adrenaline was the main catecholamine detected in the conditioned medium, suggesting that the adrenaline/noradrenaline ratio may be seen as an index of tumorigenicity. The finding that the adrenaline/noradrenaline ratio observed in the conditioned medium of non-tumorigenic MCF-10A cells was lower than that of MCF-7 cells would fit on such a hypothesis. Interestingly, in MCF-10A cells, activation of β-adrenoceptor caused an increase of the adrenaline/noradrenaline ratio to a profile similar to that observed in the tumorigenic MCF-7 cells. β_2_- and α_2C_-adrenoceptors are among the adrenoceptors more often referred to be involved in tumorigenesis and affinity of adrenaline for these receptor subtypes is higher than that of noradrenaline [17] which suggests that adrenaline may be more efficient in activating pathways involved in tumorigenesis.

The observations that, in tumorigenic breast MCF-7 cells, the β-adrenoceptor agonist isoprenaline reduces cell viability, the clonogenic ability and production of adrenaline is puzzling and seems to contradict the hypothesis that β-adrenoceptor activation promotes tumorigenesis. Nevertheless, the observation that high concentrations of β-adrenoceptor antagonists reduce cell viability, similarly to the β-adrenoceptor agonist isoprenaline, may indicate that β-adrenoceptors could be already activated by the endogenous ligand (adrenaline) and that exposure to the exogenous agonist might be causing a desensitization of β-adrenoceptors. Furthermore, α_2_-adrenoceptors are highly expressed in MCF-7 cells [21,52] and yohimbine, a selective α_2_-adrenoceptor antagonist [53,54] also reduced the viability of tumorigenic MCF-7 cells, indicating that α_2_-, like β-adrenoceptors, are also being activated by locally produced adrenaline. This hypothesis is in line with the established model of drug interaction with G-protein coupled receptors [55] and, at present, seems to be the most likely to explain why agonists and antagonists of the same receptor type are causing the same response in the same cell model. This hypothesis must be challenged in future studies as it may also contribute to explain the controversy around the role of adrenoceptors in the proliferation of cancer breast cell lines [26,52], and to understand why adrenoceptor agonists could exert their effects in concentrations that are three to four orders of magnitude lower [56] than the commonly accepted EC_50_ values [17]. 

Several epidemiological studies have shown that propranolol may be particularly effective in reducing the incidence and mortality of different types of cancers, namely breast cancer [57,58,59]. It is biologically plausible that propranolol exerts this effect not only by blocking the β-adrenoceptor-mediated tonic activation of tumorigenic cells by the adrenaline autocrinally produced but also by reducing the conditions that favor the acquisition of a tumorigenic phenotype by non-tumorigenic cells. These mechanisms would explain its activity both in metastatic [60] and early-stage breast cancer [61].

Plasma catecholamines levels increase during stressful events [62,63] but also in other physiological conditions such as perimenopause. During perimenopause, there is an increase in catecholamines plasmatic levels, and catecholamines can trigger some of these symptoms, particularly the hot flashes [5,64]. Therefore, during perimenopause women will be exposed to increased plasma catecholamines, which, in this respect, is a situation similar to life stressful events. An adrenergic contribution to tumorigenesis would explain why, in the same age interval (45–54 years), breast cancer risk is higher in perimenopausal comparatively to postmenopausal women [65], as exposure to plasma catecholamines fades away during the transition from perimenopause to postmenopause, as evidenced by the disappearance of hot flash events [5]. 

Considering the results presented, the connection between adrenergic stimulation and tumorigenesis may be more complex than a simple immediate consequence of an increase in plasma catecholamines. It may begin by a transitory effect on breast cells of plasma catecholamines released during a stressful event (step 1), which may lead to the acquisition of tumorigenic potential by predisposed cells (step 2); and by the acquisition, by the altered breast cells, of the ability to produce more adrenaline (step 3), and this autocrine and paracrine adrenaline signaling would contribute to the spread of the adrenergic tumorigenic stimuli to the cells that surround the initial cancerous niche and to cancer progression, regardless of plasma adrenaline levels (step 4), as recently shown [66]. 

## 4. Materials and Methods 

### 4.1. Drugs and Antibodies

(−)-Adrenaline bitartrate, (−)-isoprenaline hydrochloride, prazosin hydrochloride, yohimbine hydrochloride, (±)-propranolol, α-methylDOPA, Dulbecco’s Modified Eagle’s Medium/F-12 supplemented with NaHCO_3_, Dulbecco’s Modified Eagle’s Medium with NaHCO_3_ and stable L-glutamine, epidermal growth factor, human insulin, hydrocortisone, ICI 118,551, penicillin/streptomycin and HPLC-ECD standards from the highest purity available, were from Sigma-Aldrich (Sintra, Portugal). ITaq^TM^ Universal SYBR Green supermix was from Biorad (Amadora, Portugal). Foetal bovine serum was from Biochrom and L-glutamine was from Gibco (Biotecnómica, São Mamede, Portugal). 

Goat anti-mouse IgG conjugated with Alexa Fluor 488 (a11029) and mouse monoclonal anti-phenylethanolamine N-methyltransferase (MA5-25530) were from Thermo Fisher Scientific (Loures, Portugal). Rabbit polyclonal anti-β_2_-adrenoceptor (13096-1-AP) was from Proteintech (Rosemont, IL, USA). Goat anti-rabbit IgG conjugated with Alexa Fluor 594 (ab150092) and rabbit monoclonal anti-tyrosine hydroxylase (ab137869) were purchased from Abcam (Cambridge, UK). Goat anti-rabbit IgG conjugated with horseradish peroxidase (sc-2004) was from Santa Cruz Biotechnology Inc. (Frilabo, Maia, Portugal).

### 4.2. Cells and Culture Conditions

MCF-10A breast human cells (ATCC number CRL-10317 and batch number 64066742/13-12-2017) were purchased from LGC Standards (Barcelona, Spain). MCF-7 breast human cancer cells (ECACC 86012803/25-10-2017) were purchased from Sigma-Aldrich/Merck (Sintra, Portugal). MCF-7 cells were cultured in Dulbecco’s modified Eagle medium (DMEM) containing 3.7 g/L NaHCO_3_ and stable glutamine, supplemented with 100 U/mL penicillin and 100 μg/mL streptomycin and 10% heat-inactivated foetal bovine serum (FBS). MCF-10A cells were cultured in medium DMEM/F12 containing the same supplements listed for MCF-7 cells plus 3.5 μg/mL human insulin, 20 ng/mL epidermal growth factor and 0.5 μg/mL hydrocortisone. MCF-7 and MCF-10A cells were cultured in 75 cm^2^ culture flasks, at 37 °C in a humidified atmosphere of 95% air and 5% CO_2_. For cell culture maintenance, cells were routinely subcultured twice a week, treating them with 0.25% trypsin/0.025% EDTA, and kept below 90% confluence. Cells were periodically checked for mycoplasma contamination. 

Prior to each experiment, MCF-7 and MCF-10A cells were trypsinized and centrifuged at 457× *g* for five minutes at 20 °C. Viable cells, counted using trypan blue dye exclusion method, were seeded at optimized cells densities for different assays.

### 4.3. mRNA Expression by RT-qPCR

MCF-7 and MCF-10A cells were seeded in Petri dishes of 60.1 cm^2^, at initial density of 1.0 × 10^5^ and 2.0 × 10^5^ cells/mL, respectively, and incubated overnight for cell attachment. The RNeasy Mini Kit (Qiagen) was used to extract RNA, according to manufacturer’s recommendations. RNA quantification and purity were evaluated with a Synergy HT spectrophotometer (Biotek Instruments Inc., Winooski, VT, USA). As a template, 1500 ng of RNA per sample was used in the Xpert cDNA Synthesis Mastermix kit (Grisp) for reverse-transcriptase reactions. Primers design (Table S1) was accomplished with Beacon Designer Software 7 (PREMIER Biosoft). Prior to use, NCBI BLAST analysis was used to confirm primer specificity. Following PCR, primer specificity was further checked by confirming that the dissociation curve had one single peak, with an observed Tm (primer melting temperature) consistent with the amplicon length. The relative efficiency and quality of primers was evaluated using standard dilutions of cDNA.

For qPCR amplifications, 5 μL of 2 × iTaq^TM^ Universal SYBR Green Supermix, 0.25 µM of each primer and 1 μL of template cDNA were used. In all qPCR experiments, amplifications were performed in duplicate and negative controls (no template cDNA) were included. The CFX384 Touch™ Real-Time PCR Detection System (Bio-Rad, Hercules, CA, United States) was used to perform qPCRs experiments. Conditions were as follows: 95 °C for 3 min, 40 cycles of denaturation at 95 °C for 10 s and 60 °C annealing temperature for 30 s. Melting curves of the PCR amplicons were made with temperatures ranging from 55 °C to 95 °C, with increments of 0.5 °C at a rate of 10 s/step. CFX Manager^TM^ 2.0 (Bio-Rad) was used to analyze the melting curve data. The data obtained were analyzed using the method described by Pfaffl [67]. For normalization purposes, GAPDH and β-actin were used as reference genes in each analysis.

### 4.4. Western Blot

MCF-7 and MCF-10A cells were seeded in Petri dishes of 60.1 cm^2^, at an initial density of 1.0 × 10^5^ and 2.0 × 10^5^ cells/mL, respectively, and incubated overnight for cell attachment. 

After gently washing cells ice-cold PBS, total protein extraction was accomplished in lysis buffer with protease inhibitors (1 mM Na_3_VO_4_, 1 mM NaF, 1 mM PMSF, 2 μg/mL aprotinin and 2 μg/mL leupeptin). Samples were, then, homogenized with two cycles of 15 s at 5800 rpm in the Precellys Evolution Homogenizer (Bertin Instruments, Montigny le Bretonneux, France) to ensure total cell disruption, followed by an incubation period of 1 h on ice for 1 h and a centrifugation at 20.000× *g* for 45 min at 4 °C. 

Total protein concentration in the supernatant was determined using the Bradford method, using bovine-albumin as standard. Afterwards, equal amounts of protein (50 μg) were heat-denatured through boiling at 70 °C for 10 min in 6 x sample buffer [0.35 M Tris–HCl at pH 6.8, 10% sodium dodecyl sulfate (SDS), 30% glycerol, 9.3% dithiothreitol and 0.01% bromophenol blue]. Proteins were subjected to 10% SDS-PAGE (SDS-polyacrylamide gel electrophoresis) and transferred from gels onto pure nitrocellulose membranes at 25 V and 2.5 A, for 3 min using the Trans-Blot Turbo Transfer System (Bio-Rad). Membranes were blocked for 1 h at room temperature with 5% BSA in PBST (0.1% Tween 20 in PBS pH 7.4) and then probed overnight at 4 °C with primary antibody rabbit anti-β_2_-adrenoceptor (1:500) followed by secondary antibody goat anti-rabbit IgG conjugated to horseradish peroxidase (1:5000). Stain-free total protein staining was used as a loading control. Immunocomplexes were detected by an enhanced chemiluminescence system (Novex ECL, Life Technologies, Carlsbad, CA, USA) and imaged using a ChemiDoc MP Imaging System (Bio-Rad).

### 4.5. Immunocytochemistry Assay

MCF-7 and MCF-10A cells were seeded in 96-well plates, at an initial density of 1.5 × 10^4^ and 2.0 × 10^4^ cells/mL, respectively. After 24-h incubation, cells were fixed with 4% paraformaldehyde and 4% sucrose in PBS for 10 min. Nonspecific binding sites were blocked by incubating cells with a solution containing 10% FBS, 1% bovine serum albumin, 0.1% Triton X, 0.05% NaN_3_ in PBS, for 1 h. Cells were then incubated overnight at 4 °C, in a humidified atmosphere, with the following primary antibodies: rabbit anti-TH (1:150), mouse anti-PNMT (1:400) and rabbit anti-β_2_-adrenoceptor (1:100). Primary antibodies were diluted in PBS containing 5% FBS, 1% bovine serum albumin, 0.1% Triton X, 0.05% NaN_3_. Afterwards, cells were incubated for 1 h, at room temperature, with either the secondary antibodies goat anti-rabbit IgG conjugated to Alexa Fluor 594 (1:1000, for TH and β_2_-adrenoceptors) or the goat anti-mouse IgG conjugated to Alexa Fluor 488 (1:400, for PNMT). In negative controls, the primary antibody was omitted. Cell nuclei were labelled with Hoechst 33342 (5 μg/mL) for 1 min at room temperature. Images were captured with the Lionheart FX Automated Microscope (Biotek Instruments Inc.).

### 4.6. HPLC-ECD Analysis 

MCF-7 and MCF-10A cells were seeded in 24-well plates at an initial density of 3.4 × 10^4^ and 1.0 × 10^5^ cells/mL, respectively and incubated for 24 h for cell attachment. 

To screen for the presence of endogenous catecholamines, experiments were performed in the absence of FBS in order to avoid interferences from serum catecholamines, as described by Dibner and Insel [68]. Briefly, after 24-h of incubation in serum-free media, supernatants and cells were separated into distinct fractions and acidified with 2 M perchloric acid (1:10). The samples were filtered, centrifuged and frozen (−20 °C) until analysis. Catecholamines (dopamine, noradrenaline, adrenaline, α-methylnoradrenaline) and α-methylDOPA were determined by HPLC-ECD using the 3030 Reagent kit^®^ for HPLC analysis of catecholamines in urine according to the instructions of the manufacturer (Chromsystems GmbH, Munich, Germany). The HPLC system used was a Waters Alliance 2695 pump (Waters Corporation, Milford, MA, USA) with a Rheodyne loop injector and a Decade (Antec Scientific, Netherlands) electrochemical detector (ECD) with a glassy carbon electrode set to a potential of +0.75 V and +0.50 V, respectively. Empower Pro (Waters Corporation, USA) software was used to monitor the current produced. The concentrations used for the calibration curve range between 1.0 to 1000 nM (in HCl 0.1 M) for all chemical entities tested. The concentration of catecholamines presented in each sample was calculated using the calibration curve, according to the manufacturer manual.

### 4.7. Cell Viability Assay 

Cell viability assays were performed using the MTT (3-(4,5-dimethylthiazol-2-yl)-2,5-diphenyltetrazolium bromide) tetrazolium reduction assay, as previously described [69]. MCF-7 and MCF-10A cells were seeded in 96-well plates, at an initial density of 2.3 × 10^4^ or 4.0 × 10^4^ cells/mL, respectively, and allowed to attach for 24 h.

To study the effect of drugs in cell viability, FBS concentration in the medium was reduced in both cell lines considering both cell viability and minor FBS interference in cell proliferation. As such, MCF-10A and MCF-7 cells were incubated with drugs in culture medium containing either 4% or 0% FBS, respectively. Both cell lines were incubated with adrenaline (0.1, 1, 10 μM), isoprenaline (0.1, 10 μM), prazosin (1 μM), propranolol (1, 10 μM), ICI 118,551 (1 μM) and yohimbine (1 μM), alone or in combination, in parallel with the respective control (solvent), for 24 or 72 h. After drug treatment, the cell culture medium was removed and the MTT reagent (0.5 mg/mL in PBS; 100 μL) was added to each well, followed by an incubation period of three hours, protected from light. After this period, the MTT solution was removed to dissolve the formazan crystals formed and 100 µL dimethylsulfoxide was added to each well. Absorbance at 570 nm was determined in an automated microplate reader (Synergy HT, Biotek Instruments Inc.). Results were expressed as a percentage of control (non-treated). All conditions were performed in parallel triplicates or sextuplicates.

### 4.8. Colony Formation Assay

The colony formation ability was assessed using the clonogenic assay of cells in vitro [70], adapted as described previously [71]. Briefly, MCF-7 and MCF-10A cells were seeded at an initial density of 1000 cells/well, in 6-well or in 12-well plates, respectively, and allowed to attach for 24 h. Thereafter, cells were incubated with isoprenaline (10 μM), propranolol (1 μM), and ICI 118,551 (1 μM), alone or in combination, in parallel with the respective solvent (control), for 7 days. During this period, drugs were renewed after a 72 h incubation period. 

At the end of the experiment, colonies were fixed with 4% paraformaldehyde in PBS for 5 min, stained with 0.5% crystal violet for 5 min and finally rinsed twice with distilled water. Representative images of the colonies were taken using a digital camera. Quantitative changes in the clonogenic ability were determined by extracting colonies with 10% acetic acid and measuring the absorbance at 600 nm in an automated microplate reader (Synergy HT, Biotek Instruments Inc.), since a correlation between the number of colonies formed and the absorbance of the extracted dye, using ImageJ software, was achieved (data not shown). All conditions were performed in parallel, in duplicates or triplicates.

### 4.9. Statistical Analysis 

The study was designed as an exploratory study. It was assumed that 5 independent experiments would be sufficient to detect statistically significant differences in cell viability assays. In the other assays (RT-PCR, HPLC-ECD and colony formation) a minimum of 3 independent experiments were considered for the statistical evaluation. GraphPad Prism 8 software was used to design graphs and perform statistical analysis. Differences between treatments and controls were compared using one-way analysis of variance (ANOVA), followed by the post-hoc multiple comparisons Dunnett’s test, whenever applicable, or using a Student’s *t*-test, otherwise.

## 5. Conclusions

Breast cancer cells, at least those with characteristics similar to the MCF-7 cells, have the capacity to synthesize and release adrenaline. Such capacity to release adrenaline may be triggered by exposure of non-tumorigenic cells to a β-adrenoceptor agonist. Taken together, the present results bring a new perspective to understand the increase in breast cancer incidence caused by stress and during perimenopause (a period where peaks of plasma catecholamines occur) and to interpret the claimed protective effect of propranolol and similar β-blockers in breast cancer.

## Figures and Tables

**Table 1:** Concentrations of noradrenaline and adrenaline in the conditioned medium of MCF-10A and MCF-7 cells, determined by HPLC-ECD.

	Noradrenaline (nM)	Adrenaline (nM)
**MCF-10A cells**	1.78 ± 0.36	1.65 ± 0.29
**MCF-7 cells**	1.89 ± 0.51	11.02 ± 1.05 *

Conditioned media were collected after a 24-h exposure period. Experiments were carried out in foetal bovine serum-free medium, to avoid interferences from serum catecholamines. Negative controls were carried out in medium without cells and, under these conditions, neither noradrenaline nor adrenaline were detected in the medium. Shown are means ± SEM of 5 independent experiments. Significant differences from MCF-10A cells: * *p* ≤ 0.05 (Student’s *t*-test).

**Table 2:** Capacity of non-tumorigenic (MCF-10A) and tumorigenic (MCF-7) breast cells to metabolize α-methylDOPA.

	MCF-10A Cells [α-methylNA (nM)]	MCF-7 Cells [α-methylNA (nM)]
**10 µM α-methylDOPA**	165.4 ± 8.9	11.0 ± 1.1 *
**100 µM α-methylDOPA**	316.3 ± 6.7	46.1 ± 2.8 *

Conditioned media were collected after a 24-h exposure period to 10 µM or 100 µM α-methylDOPA. Negative controls consisted of culture medium without α-methylDOPA, processed in parallel. Shown are means ± SEM of 4 independent experiments. Significant differences from MCF-10A cells exposed to the same α-methylDOPA concentration: * *p* ≤ 0.05 (Student’s *t*-test). α-methylNA: α-methylnoradrenaline.

## Supplementary Materials

Supplementary materials can be found at https://www.mdpi.com/1422-0067/21/21/7968/s1.

## Abbreviations

DMEM	Dulbecco’s Modified Eagle Medium
FBS	Foetal bovine serum
ISO	Isoprenaline
MTT	3-(4,5-dimethylthiazol-2-yl)-2,5-diphenyltetrazolium bromide
PNMT	Phenylethanolamine N-methyltransferase
SDS	Sodium dodecyl sulfate
SNS	Sympathetic nervous system
TH	Tyrosine hydroxylase
β_2_-AR	β_2_-adrenoceptors
