# Supplementary material for: β-Adrenoceptor Activation in Breast MCF-10A Cells Induces a Pattern of Catecholamine Production Similar to that of Tumorigenic MCF-7 Cells
Source: Int J Mol Sci. 2020 Oct 27;21(21):7968. doi: 10.3390/ijms21217968 (PMC7672553; doi:10.3390/ijms21217968)
Supplement: Supplementary file 1 [file ijms-21-07968-s001.zip › Figure S2.pdf]

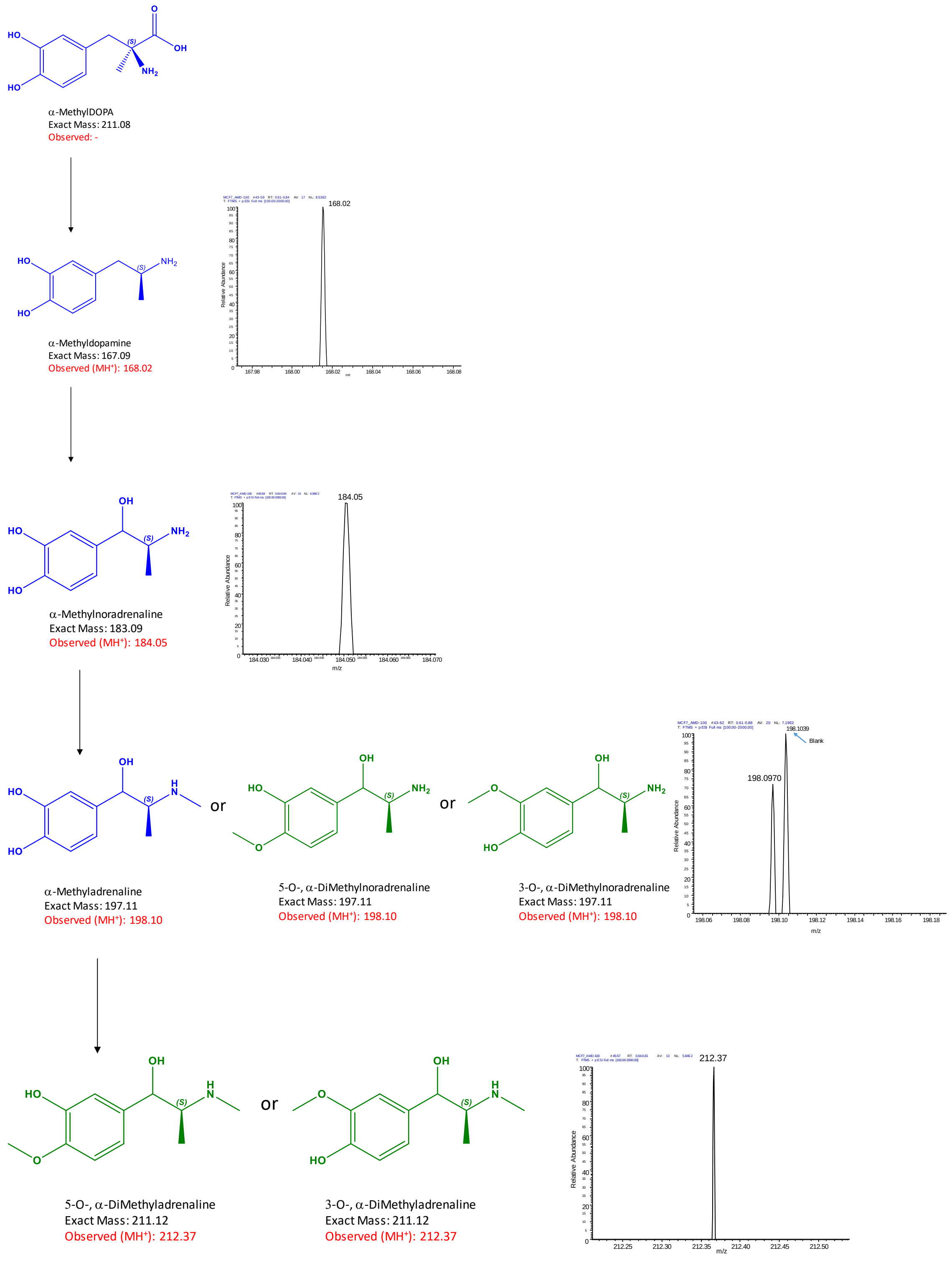

Figure S2: LC-MS identification by LC-MS of α-methylDOPA metabolites in the conditioned medium of MCF-7 cells after a 24-h incubation period. For measurement of metabolites release from MCF-7 cells, samples were separated on a HPLC Accela (Thermo Fischer Scientific, Bremen, Germany) using an ACE Equivalence 5 C18 column (ACE, Scotland) 5 µm particle size and dimensions 3.0 mm ID x 75 mm. Samples were eluted over a gradient of 100% solvent A (CH_3COOBH_4 10 mM) to reach 100% Solvent

B (ACN 0.1% HCOOH) during 8 min at a flow rate of 0.4 mL/min. Analysis were done on a LTQ Orbitrap™ XL hybrid mass spectrometer (Thermo Fischer Scientific, Bremen, Germany) controlled by LTQ Tune Plus 2.5.5 and Xcalibur 2.1.0. The capillary voltage of the electrospray ionization source (ESI) was set to 3.1 kV. The capillary temperature was 275 °C. The sheath gas and auxiliary gas flow rate were at 40 and 10 (arbitrary unit as provided by the software settings). The capillary voltage was 32 V and the tube lens voltage 55 V. MS data handling software (XCalibur QualBrowser software, Thermo Fischer Scientific) was used to search for metabolites by their m/z value.
