# Supplementary material for: β-Adrenoceptor Activation in Breast MCF-10A Cells Induces a Pattern of Catecholamine Production Similar to that of Tumorigenic MCF-7 Cells
Source: Int J Mol Sci. 2020 Oct 27;21(21):7968. doi: 10.3390/ijms21217968 (PMC7672553; doi:10.3390/ijms21217968)
Supplement: Supplementary file 1 [file ijms-21-07968-s001.zip › Figure S3.pdf]

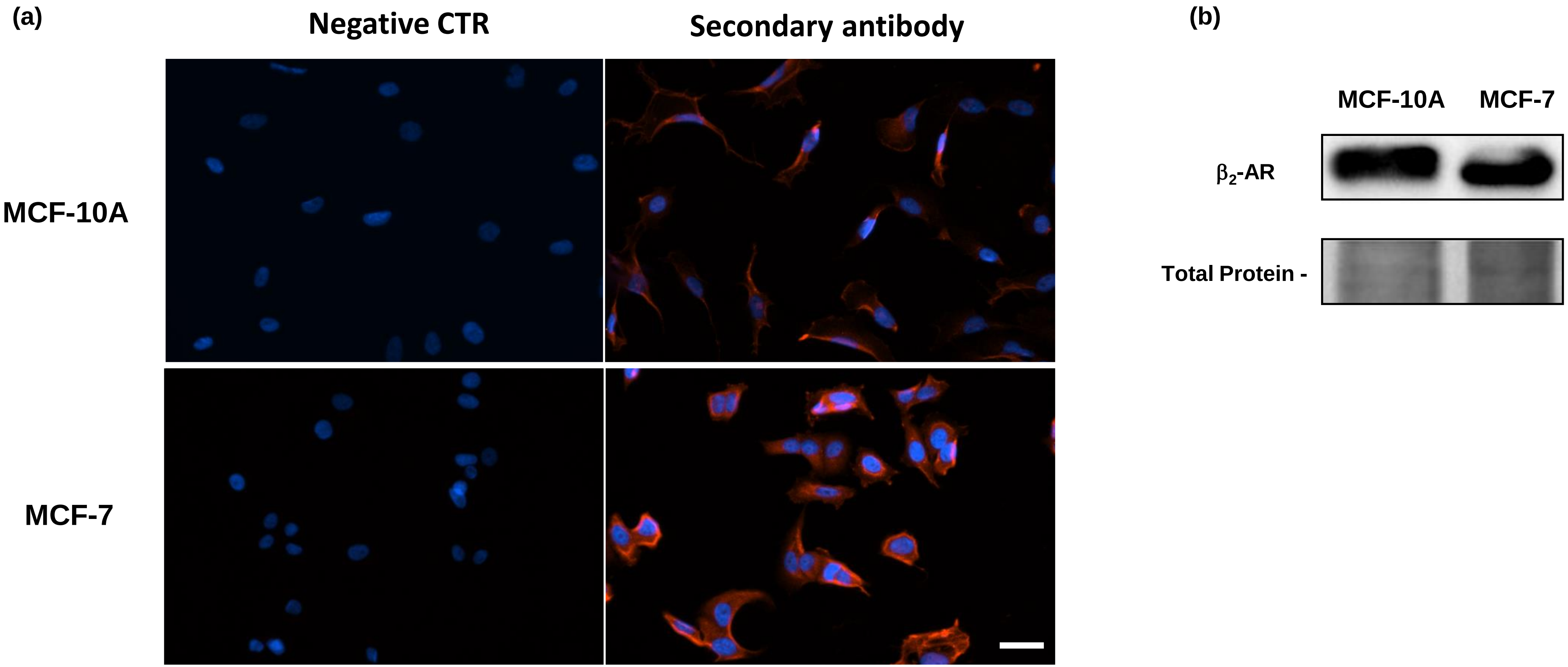

Figure S3: Expression of β_2-adrenoceptors (β_2-AR) in MCF-10A or in MCF-7 cells. (a) Representative microphotographs of MCF-10A and MCF-7 cells showing β_2-AR immunoreactivity, revealed by secondary antibody conjugated with Alexa fluor 594 (red fluorescence). Nuclei were labelled with Hoechst 33342 (blue fluorescence). Scale bar: 20 µm. Negative control (CTR): without primary antibody. **(b)** Representative western blot of β_2-AR in MCF-10A and MCF-7 cells, obtained from whole cell lysates.

Rabbit anti-β_2-AR

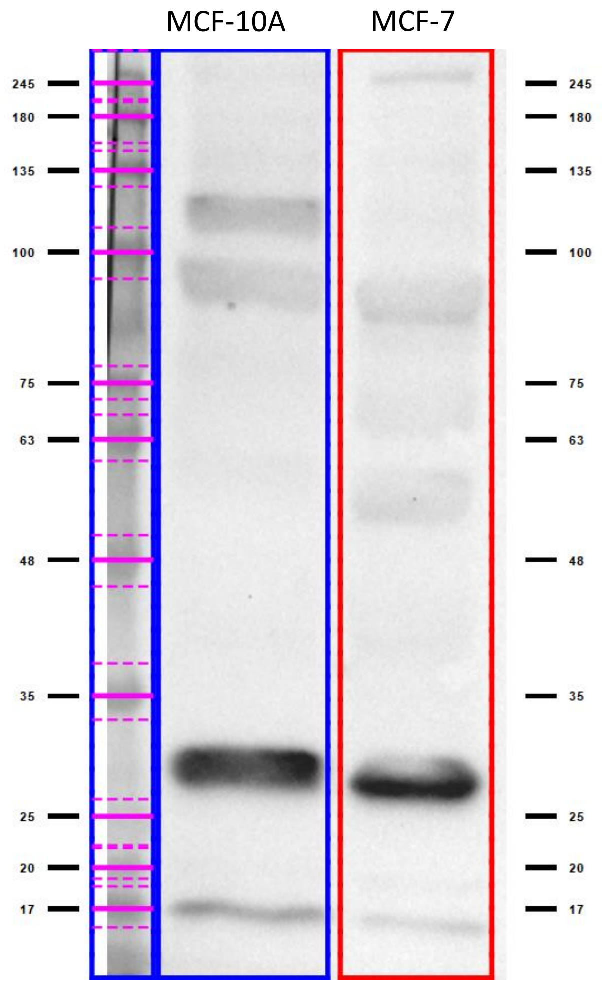

Loading control

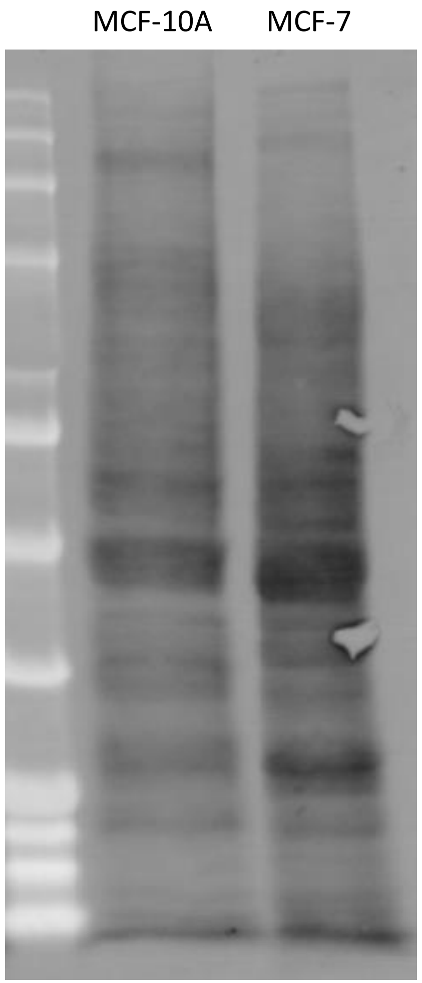

Cell line	β_2-AR	Total protein	β_2-AR/total protein
	Densitometry readings		Intensity ratio
MCF-10A	20572525,49	111568991,73	18,4
MCF-7	16884771,81	296390578,52	5,7

Western blot of β_2-adrenoceptors (β_2-AR) in MCF-10A or in MCF-7 cells. β_2-AR expression was obtained from whole cell lysates.
